# Circulating sex hormone binding globulin: An integrating biomarker for an adverse cardio-metabolic profile in obese pregnant women

**DOI:** 10.1371/journal.pone.0205592

**Published:** 2018-10-15

**Authors:** Sílvia Xargay-Torrent, Gemma Carreras-Badosa, Sara Borrat-Padrosa, Anna Prats-Puig, Pilar Soriano, Elena Álvarez-Castaño, Mª Jose Ferri, Francis De Zegher, Lourdes Ibáñez, Abel López-Bermejo, Judit Bassols

**Affiliations:** 1 Obesity and cardiovascular risk in pediatrics, [Girona Biomedical Research Institute] IDIBGI, Salt, Spain; 2 Department of Pediatrics, Dr. Trueta University Hospital, Girona, Spain; 3 Department of Physical Therapy, EUSES University School, Salt, Spain; 4 Clinical Laboratory, Fundació Salut Empordà, Figueres, Spain; 5 Department of Gynecology, Dr. Trueta University Hospital, Girona, Spain; 6 Clinical Laboratory, Dr. Trueta University Hospital, Girona, Spain; 7 Department of Development & Regeneration, University of Leuven, Leuven, Belgium; 8 Endocrinology, Hospital Sant Joan de Déu, University of Barcelona, Barcelona, Spain; 9 Centro de Investigación Biomédica en Red de Diabetes y Enfermedades Metabólicas Asociadas (CIBERDEM), ISCIII, Madrid, Spain; 10 Materno-fetal metabolic reseach, [Girona Biomedical Research Institute] IDIBGI, Salt, Spain; Mount Sinai Health System, University of Toronto, CANADA

## Abstract

Sex hormone-binding globulin (SHBG) negatively associates with pre-gestational body mass index (BMI) and gestational weight gain. The link with other cardio-metabolic risk factors in pregnant women is poorly understood. Our aim was to study the association of SHBG levels with common cardio-metabolic risk parameters in pregnant woman.

Serum SHBG was quantified in 291 Caucasian pregnant women (142 with normal weight, 42 with pregestational obesity, 50 with gestational obesity and 57 with pregestational plus gestational obesity) with uncomplicated pregnancies and parturition. Cardio-metabolic [C-reactive protein (CRP), blood pressure (BP), glycosylated hemoglobin (HbAc1), glucose, C-peptide, insulin, triglycerides and high molecular weight (HMW) adiponectin], and endocrine [testosterone and estradiol] parameters were also assessed.

SHBG was negatively correlated with BMI, but also with CRP, BP, HbAc1, pre and post-load glucose, C-peptide, HOMA-IR, triglycerides; and positively with HMW adiponectin (all p<0.01 to p<0.0001). These associations were more robust in women with pregestational plus gestational obesity, who had lower SHBG, in comparison to normal-weight women (p<0.0001). In multivariate analyses in women with pregestational plus gestational obesity SHBG showed independent associations with CRP (β = −0.352, p = 0.03, R^2^ = 8.0%), DBP (β = −0.353, p = 0.03, R^2^ = 7.0%) and SBP (β = −0.333, p = 0.04, R^2^ = 6.0%) independently of BMI and metabolic and endocrine parameters.

SHBG is decreased in pregnant women with pregestational plus gestational obesity in association with common cardio-metabolic parameters. SHBG could represent an integrating biomarker for an adverse cardio-metabolic profile in pregnant women with pregestational plus gestational obesity.

## Introduction

Obesity is a well-known worldwide epidemic condition that affects men, women and children. In recent years, much attention has been paid to obesity during pregnancy, which has not only adverse effects on the mothers’ health but also on the developing fetus [1]. Pregnant women with obesity have increased risk of impaired glucose tolerance and gestational diabetes (GDM) and increased risk of delivering a large for gestational age baby [1]. Offspring of obese women have also increased risk of obesity and obesity-related negative health outcomes later in life, such as increased carotid-intima thickness, higher body mass index, increased blood pressure or adverse lipid profile throughout childhood, adolescence, and as young adults [2,3]. Women can enter pregnancy with a body mass index (BMI) in the overweight or obese range or gain excessive weight during gestation and it is difficult to determine the separate or interdependent contributions of prepregnancy BMI and gestational weight gain on the metabolic outcomes for the mother and the offspring [4].

Adiposity is significantly associated with sex hormones, and adipose tissue contributes to the production of sex hormones in women [5,6]. Increased concentrations of sexual steroids have been related to cardio-metabolic alterations such as high blood pressure, gestational diabetes mellitus, pre-eclampsia or low/high birth weight [5,7–10]. Sex hormone binding globulin (SHBG) is a glycoprotein synthetized by the liver that transports sexual steroids (androgens and estrogens) in plasma, regulating their availability and access to target organs [11]. SHBG production is negatively regulated by insulin and monosaccharides and numerous studies in men, children and adolescents have shown that SHBG levels are reduced in obesity, insulin resistance, metabolic syndrome and type 2 diabetes [12,13]. Thus, a low level of SHBG may be a biomarker for the future development of metabolic risk factors (including hypertension, dyslipidemia, abdominal obesity and impaired glucose metabolism), and has been associated with a 2-fold increased risk of cardiovascular disease (CVD) [14]. Consistently, in postmenopausal women, low SHBG levels are related to an adverse profile of risk factors for CVD [15].

SHBG levels vary during pregnancy, being higher between 16 and 27 weeks’ gestation. The hormone is expressed in placenta as well as found in cord blood. SHBG is reduced in pregnant women with obesity and gestational diabetes [16,17]. The relationship between newborn parameters such as birth weight and maternal SHBG concentrations has been poorly investigated and is controversial, with some studies finding negative correlations and others no association [18–20]. Recent data suggest that SHBG levels during pregnancy may contribute to and predict the development of adiposity, metabolic syndrome and diabetes as children grow older [21]. If confirmed, SHBG might be a useful biomarker to detect children who are prone to develop cardio-metabolic diseases.

In summary, the link of SHBG with common cardio-metabolic parameters is poorly understood in pregnancy. We aimed to study the association of circulating SHBG with cardio-metabolic parameters in a cohort of pregnant women with pregestational and/or gestational obesity. As a secondary aim, we also studied the relationship of maternal SHBG with newborn parameters.

## Materials and methods

### Study population and ethics

The study cohort consisted of 291 mother-newborn pairs recruited among those seen at the prenatal primary care clinics in Girona, between 2008 and 2010. Inclusion criteria were: 1) singleton uncomplicated pregnancies of Caucasian origin; 2) absence of major medical, surgical or obstetrical complications; and 3) absence of maternal pathology (hypertension, pre-eclampsia or gestational diabetes). The exclusion criteria were: 1) fetal malformations or asphyxia; and 2) lack of data about principal variables. A total of 335 pregnant women were recruited in the prenatal cohort and 43 were excluded because they did not fulfil the inclusion criteria or had exclusion criteria.

Women were grouped according to their pregestational BMI and their end pregnancy weight gain following consensus guidelines from the Health and Medicine Division of the US National Academies [22], which are recommended by Spanish Society of Gynecology and Obstetrics (SEGO). The groups were as follows: 1) Normal weight women: [18.5≤pregestational BMI≤24.9 and 11.5≤pregnancy weight gain≤16kg]; 2) Women with Pregestational obesity only [pregestational BMI≥25 and 7≤pregnancy weight gain≤11.5kg or pregestational BMI>30 and 5≤pregnancy weight gain≤9kg]; 3) Women with gestational obesity only [18.5≤pregestational BMI≤24.9 and pregnancy weight gain>16kg]; and 4) Pregestational plus Gestational obesity [pregestational BMI≥25 and pregnancy weight gain>16kg or pregestational BMI≥30 and pregnancy weight gain>9kg] respectively.

The protocol was approved by the Institutional Review Board of Dr. Josep Trueta Hospital and all methods were performed in accordance with the relevant guidelines and regulations. Written informed consent was obtained from the women. All data generated or analyzed during this study are included in this published article.

### Assessments and samples

Prenatal follow-up, consisting of standardized clinical exams, ultrasonograms, and laboratory tests (urine and blood), were performed in all subjects. Social, demographic, medical and reproductive features were retrieved from the mothers’ clinical records along with labor and delivery information. Maternal education was assessed as years of schooling after primary school.

Maternal weight and height were assessed at the beginning of gestation, at second trimester and again before delivery. Maternal gestational weight gain was obtained as the difference between the last weight measurement before delivery and pre-pregnancy weight. Body mass index (BMI) was calculated as weight divided by height squared, Kg/m^2^. Systolic (SBP) and diastolic (DBP) blood pressure were measured in the sitting position on the right arm after 10 min rest; an electronic sphygmomanometer (Dinamap Pro 100, GE Healthcare, Chalfont St. Giles, United Kingdom) was used.

At delivery, placentas were weighed using a calibrated scale. Infants were weighed and measured within the first minutes after delivery using a calibrated scale for weight, a measuring board and a measuring tape for length and head circumference respectively. Gestational age- and sex-adjusted z-scores were calculated using regional norms [23]. Ponderal index was calculated as (birth weight (g)*100)/ (birthlength (cm))^3^.

### Analytical methods

All serum samples for assessment of soluble SHBG and metabolic markers were obtained under fasting conditions at second trimester of pregnancy (between 24 and 28 gestation weeks), at the time of assessment of glucose tolerance. Oral glucose tolerance tests, with fasting and one hour-timed blood glucose measurements after a 50 g oral glucose load, were performed in all participants.

Serum glucose was analyzed by the hexokinase method. HbA1c was measured by high performance liquid chromatography with ionic exchange (D-10 Hemoglobin, Bio-Rad Laboratories, Hercules, CA). Serum immunoreactive insulin was measured by immunochemiluminiscence (IMMULITE 2000, Diagnostic Products, Los Angeles, CA). Lower detection limit was 0.4 mIU/L and intra- and inter-assay CVs were less than 10%. Fasting insulin sensitivity was estimated from fasting insulin and glucose levels using the homeostasis model assessment [HOMA-IR = (fasting insulin in mU/l) x (fasting glucose in mM)/22.5)]. Serum C-peptide was measured by immunochemiluminiscence (IMMULITE 2000; Diagnostic Products, Los Angeles, CA). The detection limit was 0.05 ng/mL and CVs were less than 10%. High-molecular-weight (HMW) adiponectin was measured by sandwich ELISA (Linco, St. Charles, MO). The detection limit was 0.5 ng/mL and CVs was less than 4%. Serum levels of CRP were measured using an ultrasensitive latex immunoassay (CRP Vario; Sentinel Diagnostics, Abbott Diagnostics Europe, Milan, Italy). The lower limit of detection was 0.2 mg/L, and the intra-assay and interassay CVs were both <3%. Total serum triglycerides were measured by monitoring the reaction of glycerol-phosphate-oxidase and peroxidase. HDL cholesterol was quantified by the homogeneous method of selective detergent with accelerator. Serum alanine aminotransferase (ALT), aspartate aminotransferase (AST) and gamma-glutamyltransferase (GGT) were analyzed by colorimetry using automated tests (Roche diagnostics GmbH, Manheim, Germany). Intraassay and interassay coefficients of variation were less than 4% for these tests. Serum SHBG, estradiol and testosterone concentrations were measured by a chemiluminescent microparticle immunoassay (ARCHITECT, Abbot Laboratories SA, Texas). The within- and between-run CVs were less than 10%, and the detection limit were 0.1 nmol/L, 5 pg/mL and 0.15 nmol/L, respectively. Total testosterone was used to calculate free testosterone as previously described [24].

### Statistics

Statistical analyses were performed using SPSS version 18.0 (SPSS Inc, Chicago, IL). Results are expressed as mean ± SD for normally distributed variables and median and interquartile range for non-normally distributed variables. Kolmogorov-Smirnov test was applied to test for normal distribution. Non-normally distributed variables were mathematically transformed to improve symmetry. Differences among pregnant women groups were examined by One-way ANOVA and DMS post-hoc test or χ^2^ test. The relation of SHBG with maternal cardio-metabolic parameters at the second trimester of pregnancy (24–28 weeks of gestation) was analyzed by Pearson correlation followed by multiple regression analysis using the enter method to adjust for maternal age, maternal education, pregestational and gestational smoking, BMI, time of gestation, HOMA-IR, HMW adiponectin, hepatic enzymes, serum lipids and sex hormones. The same tests were used to study the association between SHBG at the second trimester of pregnancy and newborn parameters. Significance level was set at p<0.05.

## Results

Clinical and laboratory characteristics of the study subjects are summarized in Table 1. Women with pregestational, gestational obesity or pregestaional plus gestational obesity showed lower SHBG values than normal weight women (p = 0.05, p = 0.05 and p<0.001 respectively; Table 1).

**Table 1:** Clinical and laboratory assessments in the studied pregnant women.

	All subjects (n = 291)	Normal weight (n = 142)	Pregestational obesity (n = 42)	Gestational obesity (n = 50)	Pregestational + gestational obesity (n = 57)
Maternal Clinical assessments					
Pregestational weight (Kg)	64.4 ± 12.3	57.5 ± 6.0	78.0 ± 12.4^a^	58.8 ± 6.3^b^	76.3 ± 11.0^a^
Pregestational BMI (kg/m^2^)	24.2 ± 4.5	21.7 ± 1.9	29.5 ± 4.7^a^	21.7 ± 1.8^b^	28.8 ± 3.7^a^
Gestational weight gain (kg)	13.4 ± 5.5	11.9 ± 3.1	6.7 ± 2.9^a^^b^	20.3 ± 4.5^a^^b^	16.0 ± 4.5^a^
Gestational age at delivery (wk)	40 ± 1	39 ± 1	40 ± 1	40 ± 1^a^	40 ± 1^a^
Pregestational smoking (% yes)	31	28	36	36	34
Gestational smoking (% yes)	23	22	29	21	23
Maternal education (yr)	4 ± 3	4 ± 3	4 ± 3	4 ± 3	4 ± 3
Maternal Clinical assessments at second trimester of pregnancy:					
Age (yr)	30 ± 5	30 ± 5	31 ± 5	30 ± 5	31 ± 5
Height(cm)	163 ± 6	163 ± 5	161 ± 6	165 ± 8^a^^b^	162 ± 6
Weight(Kg)	71.4 ± 12.0	64.1 ± 6.5	81.4 ± 11.6^a^	69.4 ± 7.3^a^^b^	84.0 ± 10.8^a^
BMI (Kg/m^2^)	26.8 ± 4.3	24.2 ± 2.2	30.6 ± 4.4^a^	25.6 ± 2.2^a^^b^	31.6 ± 3.5^a^
SBP (mm Hg)	116 ± 10	115 ± 10	118 ± 10	116 ± 10^b^	121 ± 10^a^
DBP (mm Hg)	68 ± 8	66 ± 8	70 ± 8^a^	70 ± 9^a^	73 ± 8^a^
Time of gestation (wk)	26 ± 1	26 ± 1	26 ± 3	26 ± 2	26 ± 2
Maternal Laboratory variables at second trimester of pregnancy:					
Pre-load glucose (mg/dL)	79 (75–84)	78 (73–82)	81 (77–88)^a^	77 (75–83)	80 (75–85)^a^
Post-load glucose (mg/dL)	121 (97–143)	118 (97–136)	129 (99–167)^a^	110 (86–141)	125 (105–143)
HbA1C (%)	5.0 (4.8–5.2)	4.9 (4.7–5.2)	5.0 (4.8–5.3)	5.0 (4.7–5.1)	5.0 (4.8–5.3)
Fasting insulin (μIU/mL)	6.6 (1.9–8.2)	2.8 (1.1–5.7)	5.7 (3.8–12.2)^a^	4.7 (2.3–7.4)^a^	6.8 (3.6–10.5)^a^
HOMA-IR	1.3 (0.4–1.5)	0.5 (0.2–1.1)	1.2 (0.7–2.6)^a^	0.9 (0.4–1.4)^a^	1.3 (0.7–2.2)^a^
C-peptide (ng/mL)	1.5 (1.3–2.1)	1.4 (1.1–1.7)	2.0 (1.5–2.8)^a^	1.6 (1.2–2.0)^b^	2.0 (1.5–2.6)^a^
HMW-adiponectin (mg/L)	6.0 (3.7–7.7)	5.9 (4.2–8.9)	4.5 (3.4–5.5)^a^	5.9 (4.2–9.2)^b^	4.1 (3.2–6.1)^a^
CRP (mg/L)	0.5 (0.2–0.7)	0.3 (0.2–0.5)	0.6 (0.3–0.9)^a^	0.3 (0.2–0.5)^b^	0.6 (0.4–1.1)^a^
Triacylglycerol (mg/dL)	159 (116–192)	142 (116–181)	170 (121–204)	143 (101–190)	161 (131–201)
HDL cholesterol (mg/dL)	72 (63–81)	71 (63–81)	71 (59–78)	74 (64–85)	70 (63–80)
SHBG (nmol/L)	570 (463–662)	611 (490–698)	550 (440–629)^a^^b^	538 (466–662)^a^^b^	478 (386–566)^a^
AST (U/L)	14 (11–18)	14 (10–17)	14 (11–17)	15 (12–19)	14 (11–19)^a^
ALT (U/L)	15 (12–18)	14 (12–18)	14 (11–17)	15 (13–18)	15 (11–19)
GGT (U/L)	8 (7–11)	8 (7–11)	8 (7–11)^b^	8 (7–10)^b^	11 (7–16)^a^
Estradiol (pg/mL)	13884 (11034–16618)	14426 (11865–17270)	12770 (9691–15993)	13704 (9860–16735)	12292 (10445–15203)^a^
Free Testosterone (pg/mL)	0.04 (0.03–0.06)	0.03 (0.04–0.05)	0.04 (0.04–0.05)^b^	0.04 (0.03–0.06)^b^	0.07 (0.03–0.09)^a^
Newborn Clinical assessments at birth:					
Placental weight (g)	606 ± 123	568 ± 102	658 ± 134^a^	644 ± 152^a^	629 ± 118^a^
Weight (g)	3295 ± 453	3157 ± 378	3338 ± 445^a^^b^	3411 ± 497^a^	3523 ± 482^a^
Weight SDS	0.05 ± 1.04	-0.21 ± 0.83	0.09 ± 1.1	0.29 ± 1.18^a^	0.48 ± 1.2^a^
Length (cm)	49.4 ± 2.0	48.9 ± 1.9	49.4 ± 2.3	50.0 ± 2.1^a^	49.9 ± 2.1^a^
Length SDS	-0.23 ± 1.16	-0.39 ± 1.01	-0.33 ± 1.40	0.09 ± 1.16^a^	-0.06 ± 1.27
Head circumference (cm)	34 ± 1	33 ± 1	34 ± 1^b^	34 ± 2^a^	34 ± 2^a^
Head circumference SDS	-0.86 ± 1.46	-1.17 ± 1.36	-0.92 ± 1.20	-0.48 ± 1.64^a^	-0.34 ± 1.54^a^
Ponderal index (g/cm^2^)	1.3 ± 0.1	1.3 ± 0.1	1.4 ± 0.2^a^	1.4 ± 0.1^a^^b^	1.4 ± 0.1^a^

Mean ± SD or median (interquartile range). BMI: body mass index; SBP/DBP: systolic/diastolic blood pressure; HOMA-IR: homeostasis model assessment insulin resistance; CRP: C-reactive protein; SHBG: Sex hormone-binding globulin. DMS post-hoc analysis

^a^p<0.05 vs normal

^b^p<0.05 pregestational or gestational obesity vs pregestational plus gestational obesity.

In the studied women, decreasing concentrations of SHBG were correlated with a less favorable cardio-metabolic profile (more CRP, DBP, SBP, BMI, HbAc1, pre and post-load glucose, C-peptide, HOMA-IR, triglycerides and less HMW adiponectin; all p<0.05 to p<0.001; Table 2). Most of these associations were not apparent in normal weight women or women with pregestational or gestational obesity only but were present in women with pregestational plus gestational obesity (Table 2).

**Table 2:** Pearson correlation analyses of SHBG with clinical and laboratory parameters in the studied pregnant women.

SHBG	All subjects (n = 291)		Normal weight (n = 142)		Pregestational obesity (n = 42)		Gestational obesity (n = 50)		Pregestational + gestational obesity (n = 57)	
	r	P	r	P	r	P	r	P	r	P
Maternal assessments										
Pregestational weight	-0.301	<0.001	-0.155	Ns	-0.358	0.020	-0.171	Ns	-0.022	Ns
Pregestational BMI	-0.311	<0.001	-0.231	0.006	-0.256	Ns	-0.029	Ns	-0.150	Ns
Gestational weight gain	-0.098	Ns	0.055	Ns	-0.074	Ns	-0.054	Ns	0.092	Ns
Gestational age at delivery	-0.023	Ns	-0.003	Ns	0.061	Ns	0.052	Ns	0.321	0.018
BMI	-0.359	<0.001	-0.250	0.004	-0.226	Ns	0.004	Ns	-0.191	Ns
SBP	-0.175	0.005	-0.089	Ns	-0.055	Ns	-0.037	Ns	-0.419	0.003
DBP	-0.238	<0.001	-0.165	Ns	-0.065	Ns	0.004	Ns	-0.459	0.001
Pre-load glucose	-0.192	0.001	-0.082	Ns	-0.374	0.015	-0.468	Ns	-0.071	Ns
Post-load glucose	-0.156	0.008	-0.094	Ns	-0.039	Ns	-0.294	0.040	-0.098	Ns
HbA1C	-0.123	0.03	0.026	Ns	-0.343	0.026	0.012	Ns	-0.262	0.049
Fasting insulin	-0.192	0.001	-0.017	Ns	-0.229	Ns	-0.141	Ns	-0.304	0.021
HOMA-IR	-0.201	0.001	-0.022	Ns	-0.254	Ns	-0.149	Ns	-0.295	0.026
C-peptide	-0.325	<0.001	-0.144	Ns	-0.226	Ns	-0.364	0.010	-0.432	0.001
HMW-adiponectin	0.192	0.001	0.146	Ns	0.107	Ns	0.091	Ns	0.159	Ns
CRP	-0.288	<0.001	-0.149	Ns	-0.004	Ns	-0.080	Ns	-0.328	0.013
Triacylglycerol	-0.127	0.03	-0.124	Ns	0.146	Ns	-0.204	Ns	-0.111	Ns
HDL cholesterol	0.002	Ns	-0.001	Ns	-0.051	Ns	0.172	Ns	-0.146	Ns
AST	0.037	Ns	0.080	Ns	0.038	Ns	0.360	0.010	-0.067	Ns
ALT	-0.001	Ns	0.012	Ns	-0.141	Ns	0.355	0.011	-0.063	Ns
GGT	-0.264	<0.001	-0.252	0.003	-0.250	Ns	0.010	Ns	-0.238	Ns
Estradiol	0.203	0.01	0.156	Ns	0.217	Ns	0.080	Ns	0.095	Ns
Free testosterone	-0.479	<0.001	-0.368	<0.001	-0.419	Ns	-0.541	0.008	-0.412	0.019
Newborn assessments										
Placental weight	-0.125	0.02	0.099	Ns	0.217	Ns	-0.133	Ns	0.155	Ns
Weight	-0.120	0.03	-0.101	Ns	0.028	Ns	-0.112	Ns	0.394	0.003
Weight SDS	-0.077	Ns	-0.062	Ns	0.093	Ns	-0.163	Ns	0.296	0.030
Length	-0.108	0.05	-0.089	Ns	0.065	Ns	-0.170	Ns	0.479	<0.001
Length SDS	-0.021	Ns	-0.053	Ns	0.109	Ns	-0.251	Ns	0.389	0.004
Head circumference	-0.131	0.04	-0.044	Ns	-0.070	Ns	-0.066	Ns	0.129	Ns
Head circumference SDS	-0.106	Ns	-0.022	Ns	-0.005	Ns	-0.159	Ns	0.060	Ns
Ponderal index	-0.128	0.03	-0.088	Ns	-0.054	Ns	-0.013	Ns	0.173	Ns

The associations of SHBG with cardio-metabolic parameters remained significant in all subjects after controlling for maternal age and education, BMI, time of gestation, smoking, metabolic (HOMA-IR, hepatic enzymes and serum lipids) and endocrine parameters (HMW adiponectin, free testosterone and estradiol) in multiple regression analyses. CRP (β = −0.151, p = 0.009) and DBP (β = −0.129, p = 0.024) were independent predictors of SHBG levels in their respective models, explaining, together with BMI, GGT and sex hormones, 25% of SHBG variance (Table 3). In women with pregestational plus gestational obesity, SHBG showed independent associations with CRP (β = −0.352, p = 0.032, R^2^ = 8.0%), DBP (β = −0.353, p = 0.035, R^2^ = 7.0%) and SBP (β = −0.333, p = 0.046, R^2^ = 6.0%) independently of BMI and metabolic and endocrine parameters (Table 3).

**Table 3:** Multivariate linear models of SHBG as dependent variable in pregnant women according to obesity status.

SHBG (nmol/L)	All subjects (n = 291)			Normal weight (n = 142)			Pregestational obesity (n = 42)			Gestational obesity (n = 50)			Pregestational + gestational obesity (n = 57)		
	Beta	B	Sig.	Beta	B	Sig.	Beta	B	Sig.	Beta	B	Sig.	Beta	B	Sig.
BMI (kg/m^2^)	-0.143	-5.0	0.018	—	—	—	—	—	—	—	—	—	—	—	—
GGT (U/L)	-0.187	-5.3	0.001	-0.206	-6.1	0.017	—	—	—	—	—	—	—	—	—
Free Testosterone (pg/mL)	-0.266	-2254.1	<0.001	-0.275	-2426.0	0.002	—	—	—	—	—	—	—	—	—
**CRP (mg/L)**	**-0.151**	**-54.4**	**0.009**	—	—	—	**—**	**—**	**—**	**—**	**—**	**—**	**-0.352**	**-120.0**	**0.032**
R^2^			25%			15%			0%			0%			8%
BMI (kg/m^2^)	-0.173	-6.1	0.004	—	—	—	—	—	—	—	—	—	—	—	—
GGT (U/L)	-0.183	-5.2	0.002	-0.201	-5.9	0.022	—	—	—	—	—	—	—	—	—
Free Testosterone (pg/mL)	-0.268	-2273.3	<0.001	-0.270	-2376.7	0.002	—	—	—	—	—	—	—	—	—
**SBP (mmHg)**	**—**	**—**	**—**	—	**—**	—	**—**	**—**	**—**	**—**	**—**	**—**	**-0.333**	**-4.5**	**0.046**
R^2^			24%			14%			0%			0%			6%
BMI (kg/m^2^)	-0.157	-5.5	0.009	—	—	—	—	—	—	—	—	—	—	—	—
GGT (U/L)	-0.186	-5.3	0.001	-0.193	-5.7	0.026	—	—	—	—	—	—	—	—	—
Estradiol (pg/mL)	0.112	0.01	0.041	—	—	—	—	—	—	—	—	—	—	—	—
Free Testosterone (pg/mL)	-0.259	-2190.9	<0.001	-0.252	-2225.1	0.004	—	**—**	—	—	**—**	—	—	**—**	—
**DBP (mmHg)**	**-0.129**	**-2.3**	**0.024**	—	**—**	—	**—**	**—**	**—**	**—**	**—**	**—**	**-0.353**	**-6.1**	**0.035**
R^2^			25%			15%			0%			0%			7%

Three separate models are shown: one for each of the studied cardiovascular parameters. R^2^ is shown for the combined effect of the predictive variables in the model. Non-predictive variables: age, maternal educational, pregestational and gestational smoking, time at sampling during gestation (time of gestation), HOMA-IR, HMW adiponectin, AST, ALT, serum lipids.

SHBG levels showed negative associations with newborn parameters including placental weight, birth weight, birth length, head circumference and ponderal index (all p<0.05; Table 2). In pregnant women with pregestational plus gestational obesity, maternal SHBG levels correlated with birth weight SDS and birth length SDS (p<0.05; Table 2). However, these parameters were not significantly related to SHBG in multivariate analysis after adjusting for confounding variables (Data not shown).

## Discussion

SHBG levels are decreased in pregnant women with pregestational plus gestational obesity and are correlated with a less favorable cardio-metabolic profile (more CRP, DBP, SBP, insulin, C-peptide, HOMA-IR and less HMW adiponectin).

It is well known that SHBG levels decrease with increasing obesity [25] and rise with weight loss [26]. We also observed that women with pregestational obesity and/or gestational obesity showed lower SHBG values than normal weight women.

As expected from the current literature [1,11,27] negative associations were found between SHBG levels and metabolic parameters. Except for BMI, these correlations were not apparent in normal weight women but were present in women with pregestational plus gestational obesity suggesting that the insulin resistance state secondary to maternal obesity could elicit these associations. Interestingly, it appears that the combined contribution of pregestational plus gestational obesity increases the cardio-metabolic risk in pregnant women, since each factor alone elicits weak or absent associations.

Several studies have investigated the associations between SHBG and cardiovascular risk parameters including CRP and blood pressure [28]. However, these studies were mostly performed in men [29–31]. Studies on the association between androgens and CVD in women have been conducted mainly in the setting of polycystic ovarian syndrome, a condition that has been strongly associated with cardiovascular risk factors including obesity, insulin resistance, and lipid abnormalities [32]. The available studies of SHBG in pregnant women were focused in GDM [33–37]. Interestingly, low SHBG concentrations before pregnancy have also been associated with increased risk of GDM, suggesting that SHBG could be used as a biomarker for early detection of GDM [38]. However, none of these studies has assessed the potential association of SHBG and cardiovascular risk markers during pregnancy. Hence we show, for the first time, that SHBG associated with CRP and BP independently of metabolic (HOMA-IR, HbAc1, hepatic enzymes and serum lipids) and endocrine (HMW adiponectin, testosterone and estradiol) parameters in pregnant women with pregestational plus gestational obesity. Although we cannot demonstrate a direct role of SHBG in real cardiovascular risk, the clinical relevance of these data relies on the independent association of SHBG with a more adverse cardio-metabolic profile. Accordingly, SHBG levels measured in young adulthood were negatively associated with markers of subclinical CVD in a cohort study of young adult women followed for 18 yr. The associations were independent of BMI and HOMA-IR. In contrast, testosterone (either total or free) levels showed no associations with SHBG [39].

SHBG regulates the levels of active sex hormones. Sex hormones can control adipose tissue metabolism by stimulating receptors that trigger several phases of lipolysis and lipogenesis. Increased signaling by estrogens and androgens could be aimed at preparing the adipose tissue for the catabolic phase in late pregnancy in a depot-specific manner [40]. Knockout mice for estrogen receptor suffer from metabolic dysfunction together with increased adiposity, glucose intolerance, insulin resistance and endothelial alterations [41]. Although SHBG was believed to be only a transport glycoprotein, there is growing evidence suggesting that SHBG may have an independent biological function through the binding to its receptor in target tissues [42]. A possible direct effect of SHBG on diabetes mellitus was suggested by a recent report [17]. Direct effects of SHBG on the vasculature are therefore also plausible. In a study of coronary artery disease, long repeats in the *SHBG* gene promoter (the (TAAAA)n) were associated with low SHBG and with increased severity of coronary artery disease on angiography [43]. SHBG has been suggested to act through the steroid signal transduction system of cell membranes [44].

SHBG is present in the fetal circulation and in cord blood [36]. In general, low levels of SHBG have been described in newborns, followed by an increase until the end of infancy [45]. Children born at low birth weight show reduced levels of SHBG at prepubertal stages [46]. Low circulating SHBG levels in childhood and adolescence have been related to hyperinsulinaemia/insulin resistance [47]. However, the relationship between neonatal and maternal SHBG concentrations has been controversial. While no association was initially found between maternal SHBG concentrations and infant’s birth weight [19,20], a recent study reported that SHBG concentrations were inversely related to birth weight [17]. Similar results were previously obtained from SHBG measured in umbilical cord [18]. We observed that SHBG levels showed negative associations with placental weight, birth weight, birth length, head circumference and ponderal index, however, these parameters were not significantly associated with SHBG in multivariate analysis after adjusting for confounding variables. Differences in design and study populations could account for the disparity in the reported results.

An important strength of our study is the availability of a large representative population-based sample with detailed information on the cardio-metabolic profile for each individual. However, the limitations of our study also merit attention. No data about socio-economic variables of the families or physical activity of the mothers that could act as confounders was available; it would be interesting to test the contribution of these factors in future studies. The cross-sectional design does not allow us to address the temporality or cause-effect of the observed associations, thus whether elevated SHBG is causing increased cardiovascular risk or is definitely a mere consequence. In this line, future studies with long term follow-up of these women should assess whether these associations are transitory during pregnancy or permanently increase the risk of developing cardio-metabolic diseases in later life, and determine if this applies only to Caucasian population or to other ethnicities. Finally, study of the association between newborn SHBG levels at birth and maternal SHBG or maternal parameters should be considered, however not performed in this current study due to the unavailability of neonatal blood samples.

In summary, SHBG is decreased in pregnant Caucasian women with pregestational plus gestational obesity in association with common cardio-metabolic parameters. We suggest that SHBG could represent an integrating biomarker for an adverse cardio-metabolic profile in pregnant women with pregestational plus gestational obesity.

## Supporting information

S1 Appendix Raw data. (XLSX)
